# Supplementary material for: Changes in Skeletal Muscle Troponin T and Vitamin D Binding Protein (DBP) Concentrations in the Blood of Male Amateur Athletes Participating in a Marathon and 100 km Adventure Race
Source: Int J Environ Res Public Health. 2023 May 1;20(9):5692. doi: 10.3390/ijerph20095692 (PMC10178111; doi:10.3390/ijerph20095692)
Supplement: Supplementary file 1 [file ijerph-20-05692-s001.zip › ijerph-2282347-supplementary.pdf]

International Journal of
Environmental Research and Public Health

MDPI

Article

Changes in Skeletal Muscle Troponin T and Vitamin D Binding Protein (DBP) Concentrations in the Blood of Male Amateur Athletes Participating in a Marathon and 100 km Adventure Race

Jacek Borkowski [1,*] , Tadeusz Stefaniak [2] and Piotr Cych [3]

[1] Department of Physiology and Biochemistry, Wroclaw University of Health and Sport Sciences, 35 J.I. Paderewski Avenue, 51-612 Wroclaw, Poland
[2] Department of Immunology, Pathophysiology and Veterinary Preventive Medicine, Wrocław University of Environmental and Life Sciences, C.K. Norwida 31 Str, 50-375 Wrocław, Poland
[3] Department of Sport Didactics, Wroclaw University of Health and Sport Sciences, 35 J.I. Paderewski Avenue, 51-612 Wroclaw, Poland
* Correspondence: jacek.borkowski@awf.wroc.pl

Abstract: This study assessed changes in creatine kinase (CK) activity and skeletal muscle troponin T (sTnT) concentrations in the blood, to estimate the degree of muscle degradation after exercise. In addition, the concentration of vitamin D binding protein (DBP) in the blood was assessed. DBP concentrations were measured in blood as a marker for plasma load by monomeric actin. The study included marathon (MR) participants and 100 km adventure race (AR) participants, who were examined before and after the race. There was a significant (16-fold) increase in CK activity among AR participants, and a significant increase in sTnT concentration—127% in the MR group and 113% in the AR group, while there was a statistically significant decrease in DBP concentration by 14% in the AR group. In addition, it was observed that the initial concentration of DBP in both groups was in a normal range, but was lower than the average population, and the DBP concentration in the AR group was lower than in the MR group. It was concluded that exhausting physical effort such as a marathon or adventure races causes muscle damage with a far stronger influence on sarcoplasm than on filaments. The short-term and slight reduction in the concentration of DBP in blood after such efforts may be due to the appearance of monomeric actin in plasma.

Keywords: skeletal troponin T; vitamin D-binding protein; globulin Gc; marathon run; adventure race; muscle injury

Citation: Borkowski, J.; Stefaniak, T.; Cych, P. Changes in Skeletal Muscle Troponin T and Vitamin D Binding Protein (DBP) Concentrations in the Blood of Male Amateur Athletes Participating in a Marathon and 100 km Adventure Race. *Int. J. Environ. Res. Public Health* **2023**, *20*, 5692. https://doi.org/10.3390/ijerph20095692

Academic Editor: Guglielmo Duranti

Received: 28 February 2023
Revised: 7 April 2023
Accepted: 27 April 2023
Published: 1 May 2023

Copyright: © 2023 by the authors. Licensee MDPI, Basel, Switzerland. This article is an open access article distributed under the terms and conditions of the Creative Commons Attribution (CC BY) license (https://creativecommons.org/licenses/by/4.0/).

Supplementary

Table S1. Changes of biochemical parameters measured in blood of amateur marathon runners.

SAMPLE	A before			B after			C 24h after			D 48h after		
participant	CK [U/l]	sTnT [µg/l]	DPP mg/l	CK [U/l]	sTnT [µg/l]	DPP mg/l	CK [U/l]	sTnT [µg/l]	DPP mg/l	CK [U/l]	sTnT [µg/l]	DPP mg/l
1	319	48.4	358	724	78.8	322	1433	56	342	509	46.4	362
2	83	58.8	295	251	68.4	260	1109	53.2	295	716	41.6	300
3	136	57.0	421	1085	80.6	362	1866	59.7	384	627	50.1	440
4	92	71.5	364	394	101.2	346	1379	71.5	360	813	55.9	372
5	97	40.9	289	305	50.3	275	522	100	281	687	37.4	295
6	84	44.2	412	287	44	375	1244	54.4	380	686	16.0	406
7	122	39.6	348	686	40.9	285	2172	32.8	287	1256	33.0	354
8	97	41.3	372	214	47.2	357	543	60	368	341	54.4	384
9	286	41.7	421	1906	60.1	366	1384	66	385	2480	33.6	412

Creatine Kinase activity is expressed in International Units per liter [U/L]. Troponin T from skeletal muscles (sTnT) is expressed in micrograms per liter [µg/l]. Vitamin D Binding Protein (DBP) is expressed in milligrams per liter [mg/l].

Table S2. Changes of biochemical parameters measured in blood of amateur adventure race participants.

PARAMETER participant	CK A [U/L]	CK B [U/L]	sTnT A [µg/L]	sTnT B [µg/L]	DBP A [mg/L]	DBP B [mg/L]
11	198	1550	71	86	213	202
12	110	3900	63	71	213	157
13	111	1870	72	78	213	258
14	102	1820	73	86	235	174
15	197	2040	74	92	319	224
16	103	1630	83	91	224	216
17	107	2010	76	82	364	258
18	149	1650	76	77	297	295
19	161	3610	74	88	398	342

Disclaimer/Publisher's Note: The statements, opinions and data contained in all publications are solely those of the individual author(s) and contributor(s) and not of MDPI and/or the editor(s). MDPI and/or the editor(s) disclaim responsibility for any injury to people or property resulting from any ideas, methods, instructions or products referred to in the content.
